# Neonatal Hydrops Simulation Model: A Technical Report

**DOI:** 10.7759/cureus.13535

**Published:** 2021-02-24

**Authors:** Jillian Connors, Orna Rosen

**Affiliations:** 1 Division of Neonatology, The Children's Hospital at Montefiore, Bronx, USA

**Keywords:** hydrops fetalis, neonatal resuscitation, thoracocentesis, abdominal paracentesis

## Abstract

This technical report describes the creation of a model of a newborn with hydrops fetalis (HF). This model is easy to assemble, quite authentic and reusable allowing for many neonatal intensive care providers to practice rare, life-saving procedures. Learning objectives and a critical action checklist have been included to guide the simulation and add additional complexity to the scenario, if desired.

## Introduction

Hydrops fetalis (HF), or neonatal hydrops, is a rare phenomenon characterized by abnormal accumulation of fluid in at least two fetal compartments (pleural, pericardial, peritoneal, and/or skin) and is often associated with polyhydramnios and placentomegaly [1,2]. There are multiple etiologies, but HF is generally divided into two categories: immune and non-immune. While clinical presentations are variable depending on underlying etiology, neonates with HF are often critically ill at birth and require immediate resuscitation and life-saving procedures such as thoracocentesis, abdominal paracentesis, pericardiocentesis, and/or exchange transfusion. Prenatal diagnosis of HF frequently results in preterm delivery, and this complicates the management of these patients. HF is a relatively rare diagnosis, seen in 1 in 1500 to 3000 pregnancies, and many fetuses do not survive to delivery [1]. It is imperative that neonatal intensive care unit (NICU) providers master the emergent procedures that may be necessary in caring for critically ill neonates with HF. Thoracocentesis and abdominal paracentesis are uncommon procedures in the NICU, so we utilize simulation by easily modifying a neonatal resuscitation manikin such that neonatal-perinatal fellows, nurse practitioners, and physician assistants may demonstrate procedural competency.

## Technical report

**Figure 1:**
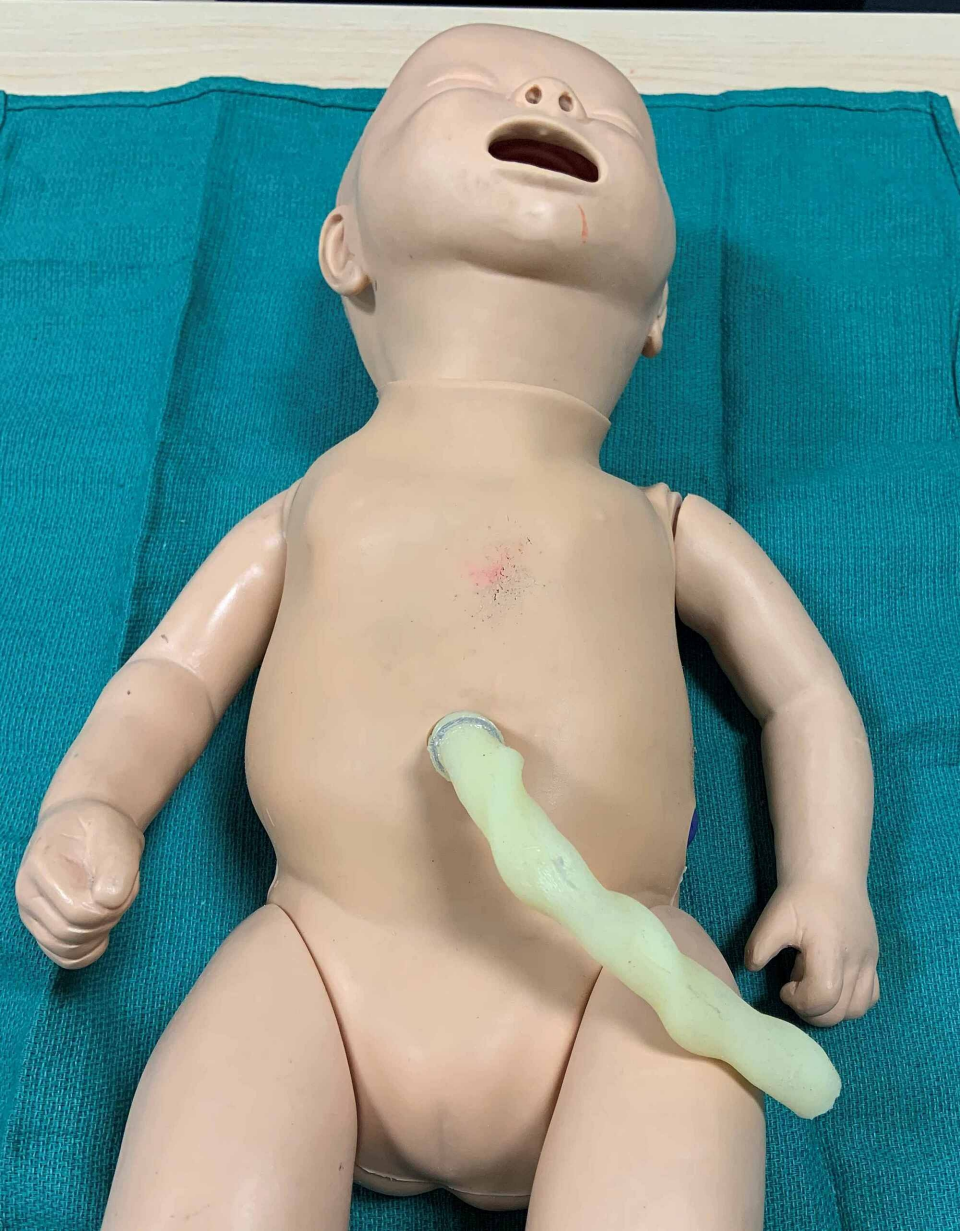
Laerdal™ neonatal resuscitation manikin (model #240-00001, Laerdal Medical Corporation, USA)

To create this pleural effusion and abdominal ascites task trainer, we modified a Laerdal™ neonatal resuscitation manikin (model #240-00001, Laerdal Medical Corporation, USA) (Figure 1). This particular model has the advantage of a removable vinyl “skin.” In addition, it has a removable rubberized shield representing the rib cage. Supplies required for manikin modification include powder-free nitrile gloves, water and povidone-iodine to simulate the color of serous effusions.

**Figure 2:**
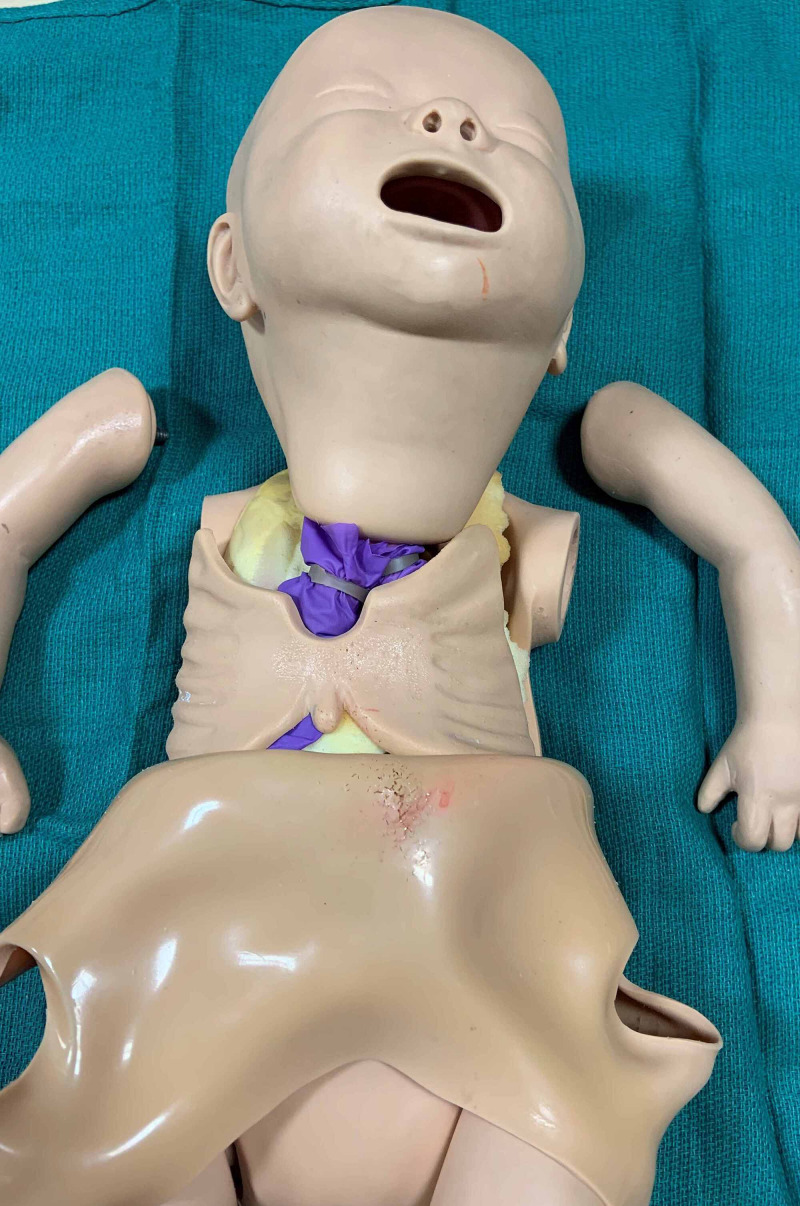
Manikin with "skin" pulled down off of shoulders after arms are removed revealing rubberized rib cage

First, the arms are removed from the manikin’s trunk. Next, the “skin” is pulled forward off the shoulders as one would doff a surgical gown (Figure 2). The rubberized rib cage and foam padding that fills the chest and abdominal cavity are then removed.

The effusions are made by filling four separate nitrile gloves with approximately 150 to 200 milliliters of water. Two to three milliliters of povidone-iodine may be added to the water to give it a straw-colored hue simulating a serous effusion. Prior to filling, the fingers of the gloves are tied together with a square knot. Once the glove is filled with water, another knot is tied at the level of the wrist.

**Figure 3:**
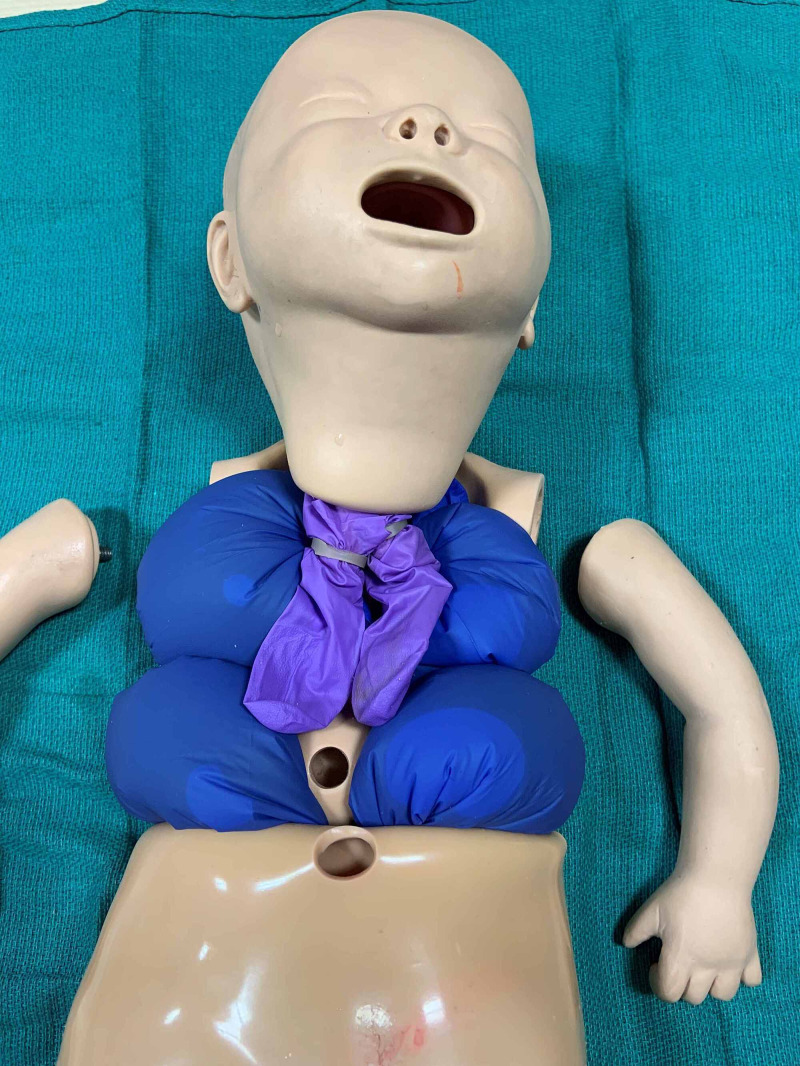
Water-filled nitrile gloves are placed in the abdominal and thoracic cavities

**Figure 4:**
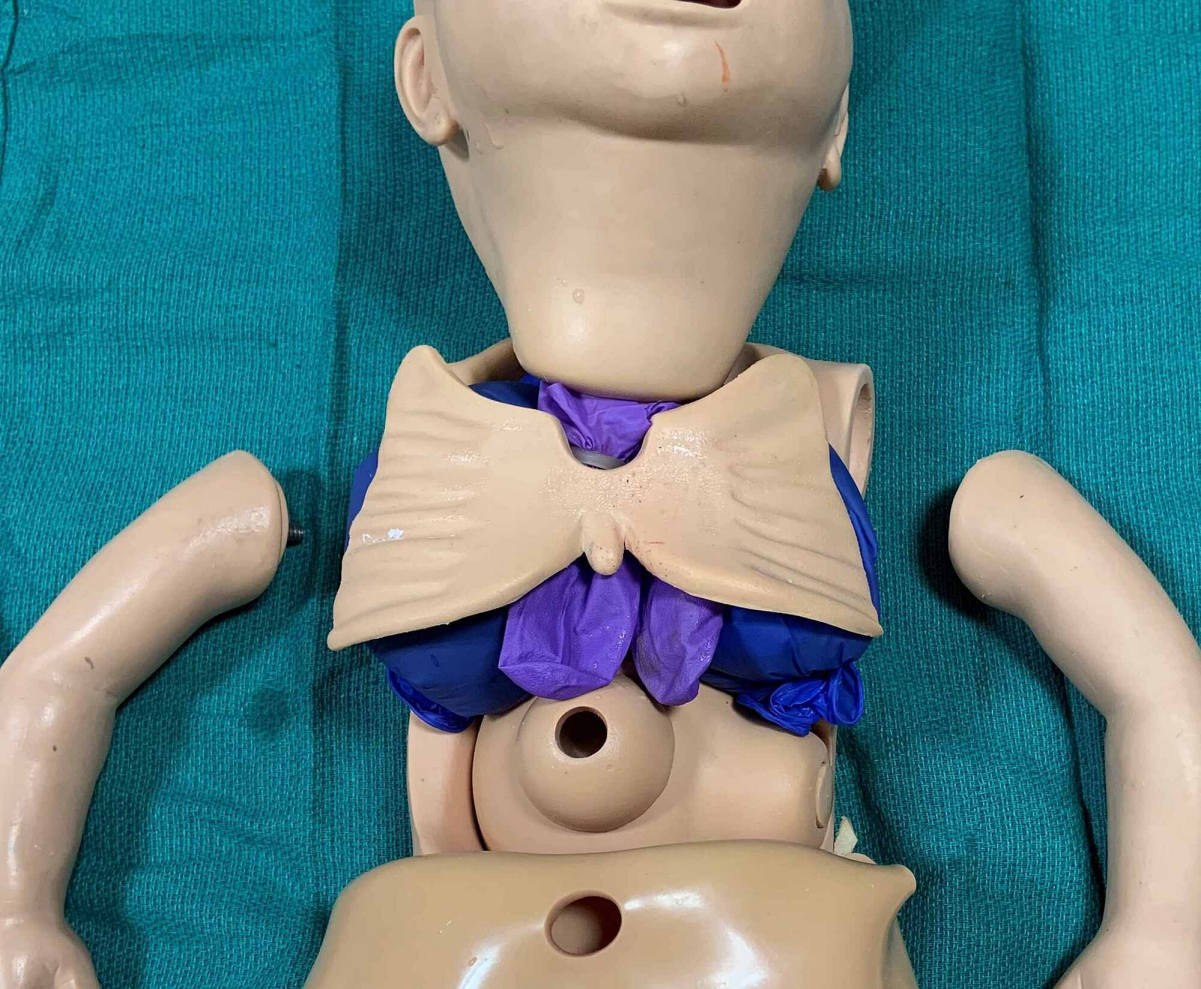
Rubberized rib cage returned to original position on top of manikin lungs

Two of the filled gloves are placed on either side of the abdominal cavity. The remaining two are placed in the thoracic cavity beneath the “lungs”, and the rubberized rib cage is returned to its original position (Figures 3-4). Please note that the foam padding is not returned to the chest and abdominal cavity. The vinyl “skin” is then pulled up over the abdomen and thorax returning it to its original position, and the arms are reattached. Additional equipment needed to perform thoracocentesis and abdominal paracentesis are outlined in Table 1.

**Table 1:** Additional equipment needed for thoracocentesis and abdominal paracentesis

Additional Equipment	
Sterile gloves and drapes	10 milliliter syringes
Povidone-iodine	Specimen containers for fluid analysis
Alcohol swabs	Petrolatum-impregnated gauze
Angiocatheters – size 20G or 22G	Gauze
Three-way stopcocks	Tegaderm or other occlusive dressing
Extension tubing or T-connector	*Optional: Tuberculin syringe with needle, local anesthetic

Technique

Emergency Thoracocentesis

**Figure 5:**
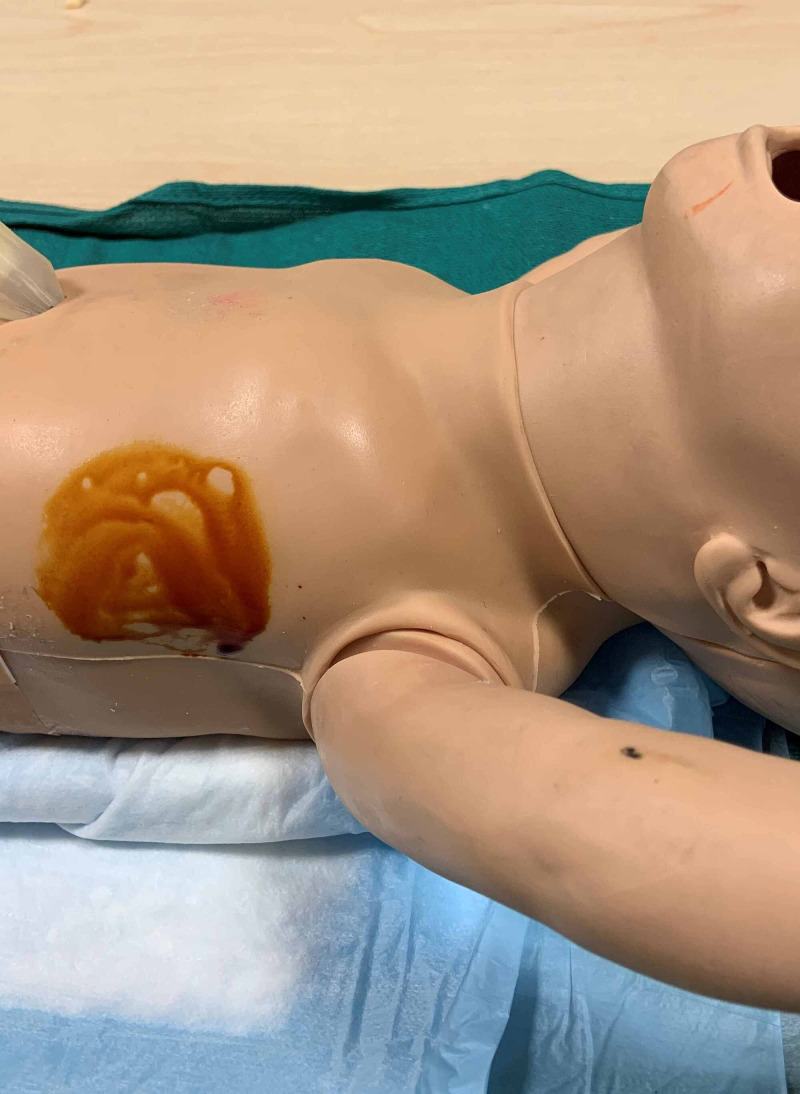
Prepped thoracocentesis site

**Figure 6:**
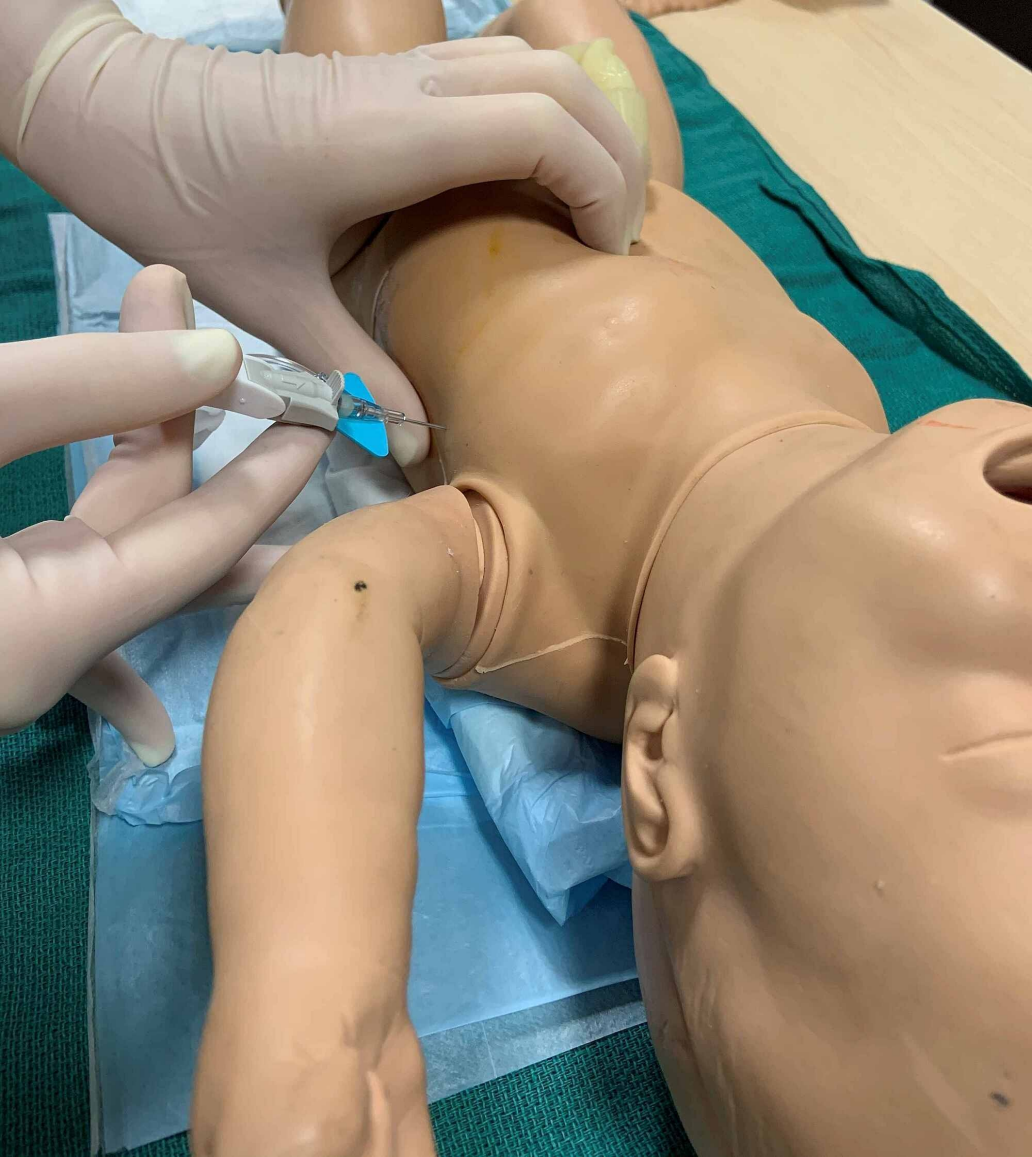
Angiocatheter insertion through chest wall into pleural space

**Figure 7:**
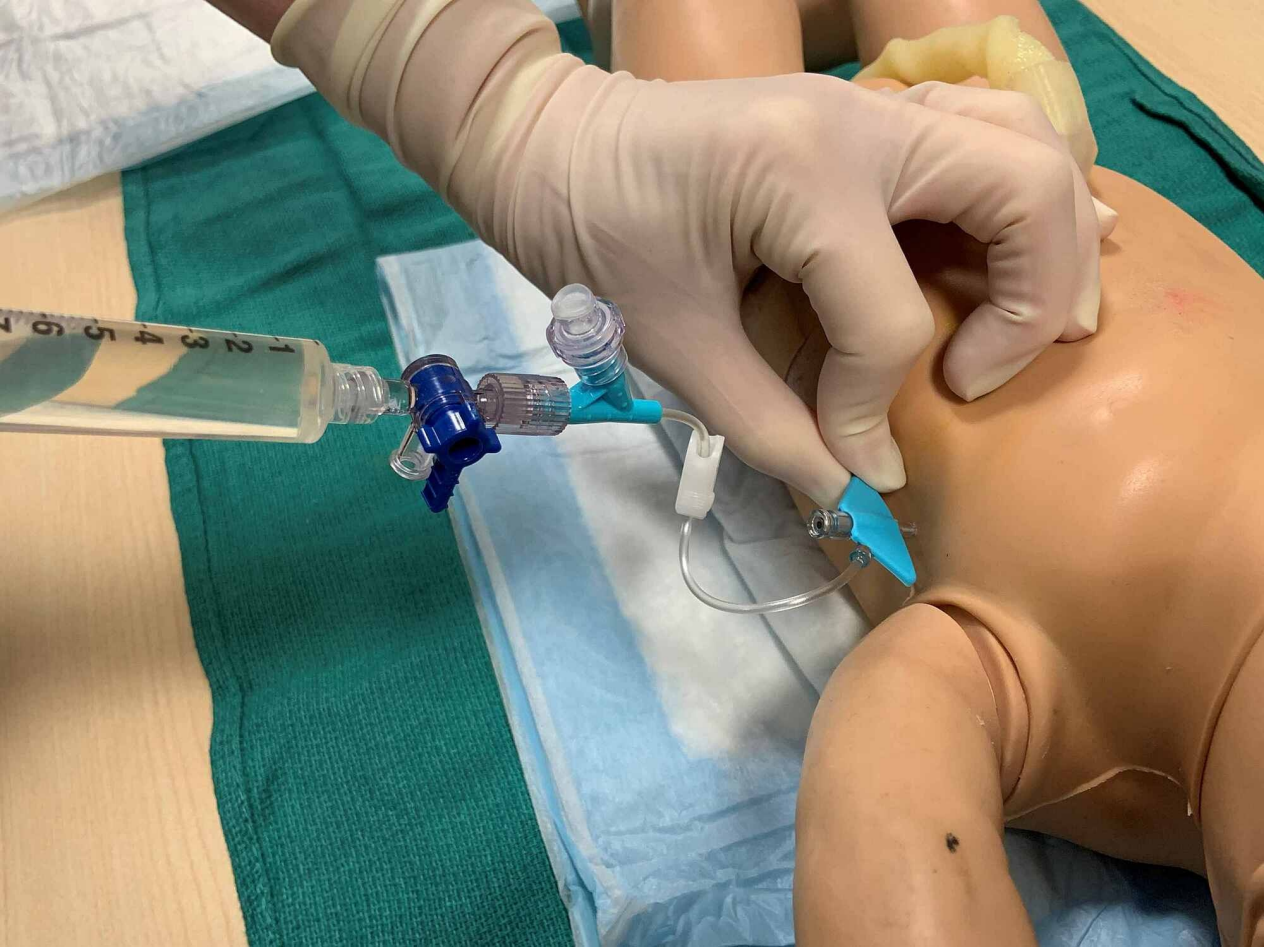
Straw-colored fluid is aspirated from pleural cavity

Thoracocentesis procedure was adapted from *Neonatology: Clinical Practice and Procedures* and *Atlas of Procedures in Neonatology* [3,4]. The patient should be in the supine position without elevation of the affected side. The procedural site is cleaned with povidone-iodine, between the anterior and posterior axillary lines at the level of the fourth and fifth intercostal space (Figure 5). Local anesthesia may be administered at the planned insertion site. A 20- or 22-gauge angiocatheter is inserted in the midaxillary line over the superior edge of the rib to avoid the neurovascular bundle and is directed posteriorly after penetrating the pleural space (Figure 6). Once in the pleural space, the needle is retracted from the angiocatheter that is then attached the extension tubing and three-way stopcock. Using a 10-milliliter syringe, pleural fluid is aspirated gently (Figure 7). Pleural fluid may be collected in a sterile container and sent for analysis, including cell count, culture and chemistry. Once no more fluid can be aspirated or oxygenation and ventilation have improved, the angiocatheter is removed, and the site should be cleaned and covered with petrolatum-impregnated gauze and an occlusive dressing.

Abdominal Paracentesis

**Figure 8:**
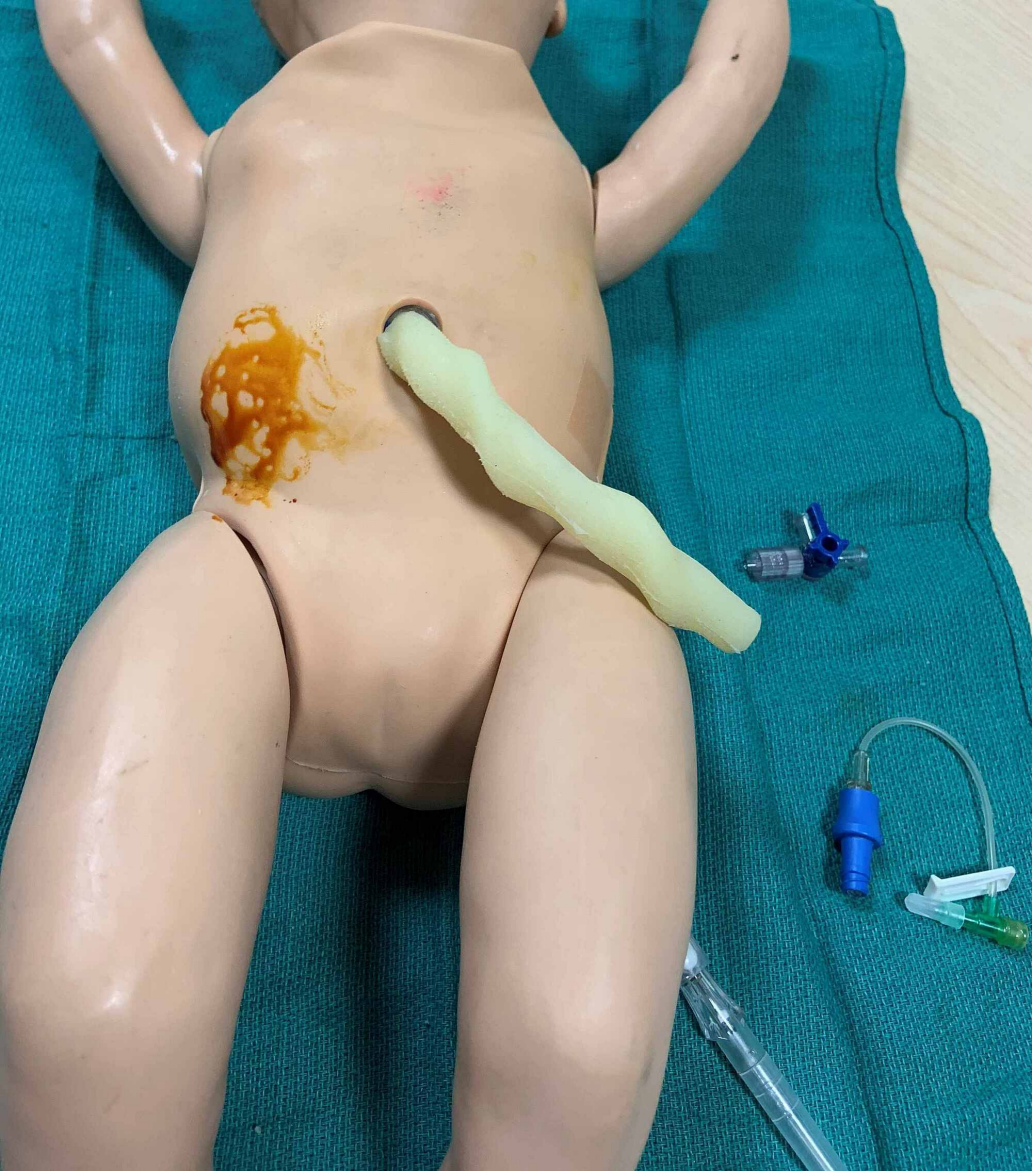
Right lower quadrant is prepped for abdominal paracentesis

**Figure 9:**
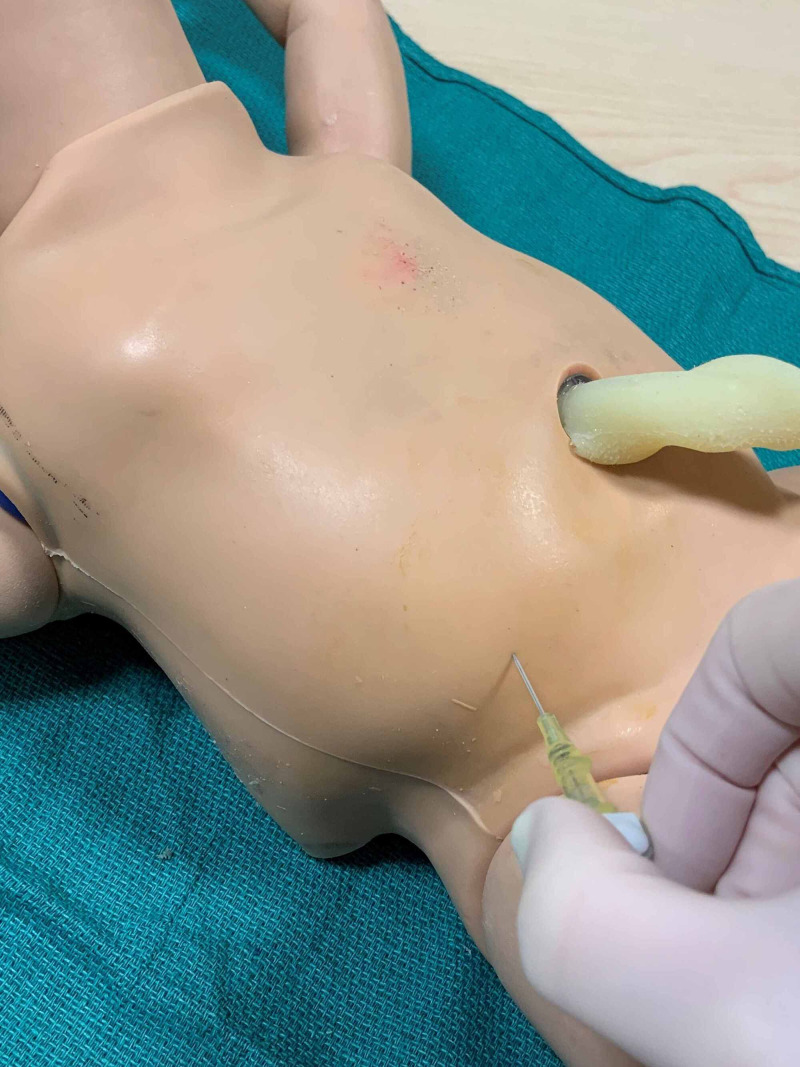
Site of angiocatheter insertion two-thirds the distance between umbilicus and anterior iliac spine

**Figure 10:**
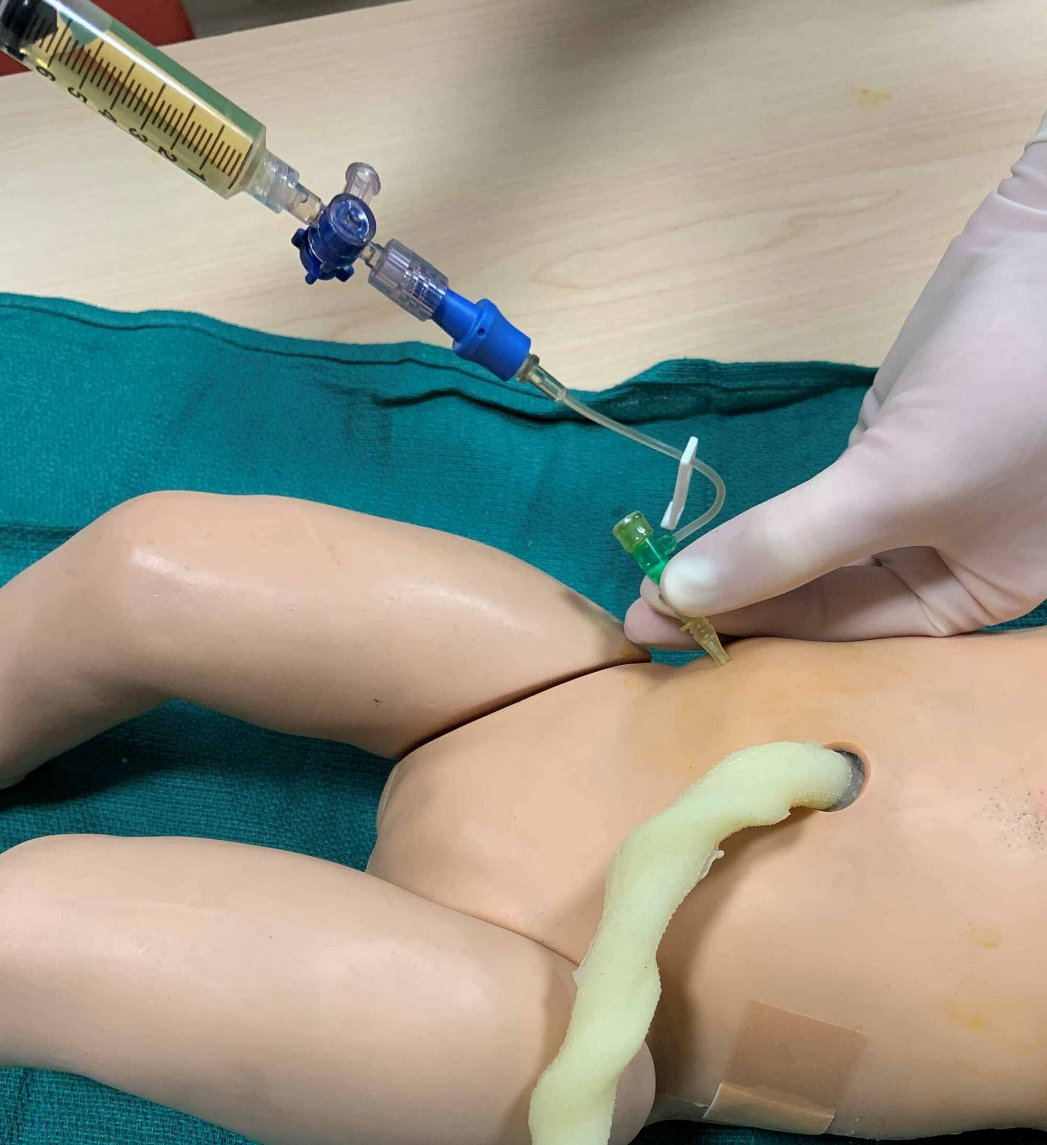
Straw-colored ascitic fluid is aspirated from the peritoneal cavity

Abdominal paracentesis procedure was adapted from *Neonatology: Clinical Practice and Procedures*, *Atlas of Procedures in Neonatology* and *Gomella’s Neonatology: Management, Procedures, On-Call Problems, Diseases, and Drugs* [3,5,6]. Again, the patient is placed in the supine position. The procedural site is cleaned with povidone-iodine (Figure 8). Paracentesis may be performed at the right lower or left lower quadrants. However, if there is concern for significant hepatomegaly, the left lower quadrant is the preferred site. The site of puncture is located two-thirds the distance along a line drawn from the umbilicus to the anterior iliac spine (Figure 9). Local anesthesia may be administered at the planned insertion site. A 20- or 22-gauge angiocatheter is inserted at a 45-degree angle and is directed posteriorly until the peritoneum is penetrated. Ascitic fluid should be visible in the angiocatheter chamber. The needle is retracted from the angiocatheter that is then attached to extension tubing and a three-way stopcock. Ascitic fluid is then gently aspirated using a 10-milliliter syringe (Figure 10). Fluid should be removed only until ventilation becomes easier in order to avoid large fluid shifts that may result in hypotension. Ascitic fluid may be collected in a sterile container and sent for analysis, including cell count, culture and chemistry. At the completion of the procedure, the angiocatheter is removed, and the procedure site should be cleaned and covered with petrolatum-impregnated gauze and a bandage with gentle pressure until leakage ceases.

## Discussion

Since the development of this task trainer at our institution, its use has been incorporated into the neonatal-perinatal fellow curriculum and NICU nurse practitioner and physician assistant continuing education. As thoracocentesis and abdominal paracentesis are relatively infrequent procedures in the NICU, it is critical that neonatal providers have ample opportunity to acquire and strengthen their technical skills. Basic learning objectives to utilize with this model are provided in the Appendix. Feedback from fellows and advanced practice providers elicited through facilitated debriefing has been overwhelmingly positive; they express feeling more confident in performing thoracocentesis and abdominal paracentesis, feel more comfortable managing HF in general and frequently request HF cases for future simulation sessions.

This model has several benefits. Not only is it a task trainer that accurately and anatomically simulates thoracocentesis and abdominal paracentesis and provides immediate feedback when the procedure is done correctly (ability to aspirate pleural effusion and ascitic fluid), but it also allows learners to practice neonatal resuscitation according to Neonatal Resuscitation Program (NRP) guidelines, including positive-pressure ventilation, intubation and/or chest compressions. This provides the opportunity for increasingly complex simulated delivery room and neonatal scenarios. Also, the model is mobile such that it can be set up in the NICU for in situ simulations or at a satellite location that is felt to be a non-threatening, protected learning environment. At the completion of the simulation, the manikin is easily cleaned and returned to its original condition.

Utilizing the critical action checklist provided in the Appendix, complexity may be added to the HF scenario. As neonates with HF are often critically ill at birth, the scenario may be adjusted such that the infant is unstable at time of delivery and requires emergent thoracocentesis and abdominal paracentesis in the delivery room, where supplies and additional support may be less readily accessible. In this scenario, the learners are required to perform resuscitation according to NRP guidelines, identify lack of improvement in ventilation and oxygenation despite following the appropriate steps and perform emergent thoracocentesis and abdominal paracentesis to achieve a level of stability appropriate for transporting the patient to the NICU. In addition, as fetuses with HF are frequently delivered preterm due to worsening status, adding complications of preterm delivery, such as respiratory distress syndrome or pulmonary hypoplasia, will increase complexity and broaden the learning objectives of the simulation [1]. The scenario may also be adjusted based on specific etiologies of HF. In the case of immune HF, learners must recognize severe anemia and manage it by calculating the appropriate volume of packed red blood cells needed for exchange transfusion. Cardiovascular etiologies, including structural heart disease and congenital arrhythmias, are the leading cause of nonimmune HF, and may be added to the scenario to direct learners to perform additional work-up, such as obtaining an electrocardiogram or echocardiogram [2].

Apart from the technical aspects of the scenario, a critical component for learners, particularly neonatal-perinatal fellows, is to practice providing a parental update. HF has a high risk of mortality, and being able to provide a complex medical update while also conveying the critical and often grave condition of a newborn is as important as being able to successfully perform thoracocentesis or abdominal paracentesis [2].

At the completion of the scenario, neonatal fellows and advanced practice providers are directed to debrief the medical and technical aspects of the simulation. Neonatal-perinatal medicine faculty facilitate the debrief using the critical action checklist as a guide and provide additional feedback to ensure that learning objectives have been met.

## Conclusions

This technical report describes the modification of a neonatal resuscitation manikin into a task trainer for thoracocentesis and abdominal paracentesis. This model is easily assembled, cost-effective, authentic, and reusable. With the described specifications, neonatal intensive care providers can utilize this model to gain more experience and confidence in performing life-saving procedures that may otherwise be infrequently performed in the NICU. In addition, this task trainer can be incorporated into existing simulation curricula to add complexity to neonatal resuscitation scenarios and expand educational opportunities for many levels of learners.

## Appendices

Appendix 1

Learning Objectives

1. Describe and perform delivery room and early neonatal management of a neonate with hydrops fetalis.

2. Gather supplies needed for urgent/emergent thoracocentesis and abdominal paracentesis

3. Correctly perform thoracocentesis and abdominal paracentesis

Appendix 2

Critical Action Checklist for Hydrops Fetalis Scenario

1. Ensure adequate supplies are accessible, including uncrossed, unmatched Type O blood in the setting of immune hydrops or severe anemia.

2. Delegate tasks to team members

3. Provide initial steps according to Neonatal Resuscitation Program guidelines.

4. Intubate the neonate

5. Transfer to NICU when medically “stable,” i.e. if oxygen saturation and heart rate are within reasonable ranges

6. Place umbilical venous and arterial catheters if the neonate is critically ill. Send blood gas, complete blood count, blood type and screen, electrolytes, and liver function tests

7. Order chest and abdominal X-ray to assess position of endotracheal tube and umbilical catheters and to assess for effusions.

8. Identify when pleural effusions require drainage, perform the procedure correctly and send proper testing on pleural fluid. Describe potential complications of the procedure.

9. Identify when abdominal ascites require drainage, perform the procedure correctly and send proper testing on ascitic fluid. Describe potential complications of the procedure. Consider obtaining abdominal ultrasound prior to procedure as dictated by clinical scenario.

10. Recognize and treat severe anemia. Calculate the volume of packed red blood cells necessary to increase hematocrit to 30%. Describe how to perform an exchange transfusion.

11. Identify need for vasopressors and/or inotropes (hypotension)

12. Obtain echocardiogram to evaluate cardiac structure and function and for pericardial effusion.

13. If hypoxemic respiratory failure is apparent, calculate oxygenation index and initiate inhaled nitric oxide therapy, if indicated.

14. For preterm infants, consider giving surfactant if there are chest X-ray findings consistent with respiratory distress syndrome.

15. Provide a summary and medical update to the patient’s parents.
